# Social Enterprise, Population Health and Sustainable Development Goal 3: A Public Health Viewpoint

**DOI:** 10.5334/aogh.3231

**Published:** 2021-06-24

**Authors:** Glória Macassa

**Affiliations:** 1 Department of Public Health and Sports Sciences, University of Gävle, 801 76 Gävle, Sweden; 2 EPIUnit – Instituto de Saúde Pública, Universidade do Porto, Rua das Taipas 135, 4050-600 Porto, Portugal

## Abstract

Although there is no consensus on the definition of “social enterprises (SEs),” various scholars have agreed that SEs are “sustainable ventures that combine business principles with a passion for social impact.” Using a public health lens, this viewpoint paper attempts to discuss the potential role SEs might play in the achievement of sustainable population health and Sustainable Development Goal 3 (SDG 3): “Health for all at all ages.” Through their impact on social determinants of health (the conditions in which people are born, grow, work, and age), SEs have a potential to contribute to SDGs, specifically SDG 3. They can do so by acting on and modifying the economic, social and environmental challenges communities face, to help promote health and wellbeing and improve the quality of life among children, adolescents, working adults and elderly across countries, societies and generations. Social enterprises present an opportunity to engage business as partners in health promotion – which is yet to materialize in all societies globally.

## Introduction

Although there is no consensus on the definition of social enterprises (SEs), various scholars have agreed that SEs are sustainable ventures that combine business principles with a passion for social impact [1,2,3,4]. For instance, it is posited that SEs strive to create social value as a primary organizational objective by employing business concepts to sustain their operations in pursuit of this objective [5]. Furthermore, others have positioned SEs on a map of organizational forms relative to the ways organizations plan to implement social change and the [degree] to which they apply business practices to do so [6,7].

Although there are differences in SE activity across countries, SEs have grown across the world in recent decades as they attempt to address social needs not addressed by the government and/or the trade sectors [8]. For instance, in some countries, SEs are essential for employment creation; in others, SEs have emerged as a result of non-governmental organizations’ (NGOs) activities to alleviate poverty, strengthen education and facilitate job creation in informal socioeconomic conditions [8].

The increased interest in SEs worldwide is based on the role they play in addressing unsolved social problems on an international scale while enhancing human development around the world and improving quality of life [8,9,10]. For instance, SE is recognized as a powerful tool to reduce unemployment [9], control poverty [8,10], address environmental issues and empower women [11,12]. This has encouraged SE to flourish around the world, especially in societies where these problems are more prominent [13].

Social enterprises have been studied extensively within the disciplines of management and marketing, and most recently much attention has been paid to how they enhance individuals’ and communities’ wellbeing. Furthermore, within the same disciplines, researchers have attempted to connect SEs to sustainable development goals (SDGs). However, this discussion has been absent in the context of population health, and SDG 3 in particular (“to ensure healthy lives and promote wellbeing for all at all ages”). Therefore, using a public health lens, this view point paper attempts to discuss the potential role SEs might play in the achievement of sustainable population health and SDG3. Firstly, the viewpoint describes the relationship between SE and sustainability; secondly, it investigates the link between SEs, population health and SDG 3; and thirdly, it discusses potential opportunities and challenges that SEs might face as they attempt to solve society’s most wicked problems, and may ultimately contribute to sustainability, population health and wellbeing.

## Social Enterprise and Sustainability

In recent years, various authors have drawn attention to the potential relationship between social entrepreneurship and sustainability [14,15,16,17]. Sustainability has three dimensions which are closely linked to each other – economy, the environment, and society [17]. The economic dimension of sustainability relates to the provision of goods and services for human needs while minimizing environmental damage. It includes production processes, consumption, and distribution [2]. The environmental dimension is about preserving natural processes, meaning that the diversity of species is maintained; also, natural resources are maintained for biological systems, and the natural and human society have self-cleansing properties [2]. Lastly, the social dimension of sustainability is related to the preservation of values such as peace, liberty and equity, which contribute to healthy societies.

It is argued that a sustainable society is one that aims at eradicating unequal social structures through improvements in redistributive policies related to income, and promotion of equality of opportunity for all and especially the most disadvantaged groups in society [2]. Linked to sustainability are the SDGs which can be understood to represent a myriad of complex global challenges that require a large number of innovations to address them [2,17].

Social enterprises can play an important role in helping to address the complex societal challenges included in the SDG agenda [14,15,16,17,18,19,20,21,22,23,24,25]. It has been suggested that SEs can help respond to societal challenges through: (1) development or adoption of a (partial) solution (i.e. social innovation); and (2) making sure that the solution is accessible (i.e. scaling of social innovation) based on a viable business model [17]. Others have argued that SEs can deliver social impact through business models that use principles of circular economy, with a focus on social and environmental problems, contributing to cost saving, new forms of revenue, resource conservation, and long-term competitiveness, as well as driving for sustainable development [26]. Furthermore, SEs are expected to contribute to SDGs by generating positive social and environmental impacts throughout their value chains. Holt and Littlewood suggest that this could occur during the input stage, for example through ethical sourcing of products. Moreover, in an SE’s operations, the SE may employ individuals from marginalized populations; or generate a positive social impact through the products and services they offer, or through direct programmes and interventions [27].

Recently, Littlewood and Holt proposed a framework relating SEs to SDGs [28]. In their novel model linking SEs to SDGs they listed four types of SEs: (1) SEs that are focused contributors (i.e. whose contributions are concentrated in a particular area of their value chain – in this instance, profits or surpluses, which are donated to charitable causes – and are narrowly focused on one SDG or a few SDGs) [28]; (2) SEs that are focused integrated contributors (i.e. that focus on contributing to one or a relatively small number of SDGs); (3) SEs that are broad contributors (whose impacts on the SDGs stem mostly from a particular aspect of their value chain – in this case, their profits or surpluses, which are donated – but that contribute to many different SDGs simultaneously); and (4) SEs that are broad integrated contributors, which includes SEs that contribute to a variety of SDGs across multiple value chain activities [28].

## Social Entrepreneurship, Population Health and Sustainable Development Goal 3

Various scholars have argued that SEs can help address the social determinants of health towards the achievement of health equity [29,30,31,32,33], which in turn will contribute to attainment of the SDGs and, specifically, SDG 3. This may occur as an upstream intervention through a “set of actions with a coherent objective to bring about change or produce identifiable outcomes [34].”

Social enterprises can reduce inequalities in health (through social, economic and/or environmental action on social determinants of health) in a variety of contexts. “Social determinants of health” are the conditions in which people are born, grow up, live, work, and age, which are crucial to people’s health. It is argued that no matter how good the local health system, access to that system and the lifestyles of individuals are dependent to a very large extent upon factors in the social environment. Rather than acting on individual risk factors such as smoking, alcohol, diet, and exercise (a pathogenic approach to health), SEs address inequities more broadly by acting on the social, economic and environmental circumstances of the most vulnerable members of society [29,30,31,32].

In 2008 the World Health Organization (WHO)’s Commission on Social Determinants of Health (CSDH) pointed out that the social distribution of health is not a natural phenomenon but, rather, the result of a toxic combination of poor social policies and programmes, unfair economics, and bad politics [35]. They made three important recommendations for reducing the persistent and widening inequities: (1) improve daily living conditions; (2) tackle the inequitable distribution of power money and resources; and (3) measure and understand the problem and assess the impact of action [35].

It has been pointed out that the social mission of SEs can be framed in terms of the “assets” they look to create, enhance, and improve – both within individuals and within the communities in which these individuals live [29,30]. Furthermore, it is suggested that, as SEs look upstream and through their work employ initiatives that utilize and build upon existing collective resources or on the “assets” that individual and communities already have at their disposal to promote health and wellbeing, they change the conditions that lead to adverse health behaviours rather than simply focusing on their deficiencies [29,36]. This has been referred to as an “assets-based” approach to public health [37,38,39,40]. Improving daily living conditions through SEs will entail creating, enhancing, and improving physical, mental and social wellbeing, focusing particularly on enhancement of individuals’ sense of coherence [41,42]. By “sense of coherence” is meant the skills and confidence to manage the demands of life, to respond to an environment that is both comprehensible and manageable [41,42].”

Moreover, SEs (and the social economy) can play a significant role in building and maintaining social capital (networks, together with shared norms, values and understandings, that facilitate co-operation within and among groups) [43,44,45]. Across the world, SEs can also alleviate poverty and provide care to the most disadvantaged groups in society, which in turn improves health, wellbeing and quality of life [46,47]. However, the majority of studies that have investigated the relationship between social enterprise and health (e.g. women and elderly) using the upstream approach (intervening on the social determinants of health) have been carried out in high income countries (e.g. UK, Australia and United States) [30,31,32,33].

Social enterprises are also considered to contribute to health promotion and population health by themselves being a healthy workplace [48,49]. Although scarce, there is evidence pointing to a significant association between social enterprises and components of “good” working conditions. Examples of providing “good” working conditions are enabling workers to exert some control through: participatory decision making on, for example, the place and timing of the work, and what tasks to do and how to accomplish them; placing appropriate high demands on the worker; providing adequate support at work; providing sufficient job security; offering opportunities for both professional and personal development; and giving workers the possibility to reconcile work and extra-work/family demands [48]. In addition, SEs are seen to offer job satisfaction; guarantee fair pay; prevent social isolation, any form of discrimination, and violence; enable their workers to share relevant information within the organization; and attempt to reintegrate sick and disabled people into employment [48,49].

Empirical evidence has shown that adverse psychosocial work environments, or a lack of “good” work in terms of unmanageable demands placed on the employee and inadequate control and support provided to them, are associated with a number of socially and economically costly health problems, including mental health problems such as anxiety and depression, and physical health problems such as musculoskeletal disorders (MSDs) and cardiovascular diseases [50,51,52,53,54,55,56]. For instance, some point to the participatory nature of the work of SEs that seek to involve employees in decision-making procedures which in turn provide supportive work environments that benefit workers [55,56,57]. Allowing employees to exert control through participatory decision making and providing them with adequate support are two important determinants of “good working conditions” thought to positively impact on employee health and wellbeing [58,59,60,61,62]. Others argue that SEs might provide “good work” as they exist to improve the lives of individuals and communities and many seek to do this by providing employment [29,63], often actively employing people from the communities they are set up to improve (e.g. deprived communities) and, in the case of social firms (a particular type of SE), those disadvantaged in the labour market [64,65]. Therefore, the social mission of SEs may serve as an incentive to provide working conditions conducive to employee health and wellbeing [48].

In recent decades, the workplace, particularly the psychosocial work environment, is increasingly being considered by policy makers as an important intervention point at which health can be improved and health inequalities can be reduced [66,67,68]. Therefore, the availability of “good work” could help improve population health and contribute to attainment of SDG 3 (and SDG 8) and, in part, address social gradients in the health outcomes associated with adverse psychosocial work conditions (e.g., depression and anxiety) which are costly for many organizations as well as for society as a whole [69].

## Discussion and Conclusion: Opportunities and Challenges

Through their impact on social determinants of health at society level as well as in the workplace, SEs have the potential to contribute to SDGs, specifically SDG 3. This can occur by acting on and modifying the economic, social and environmental challenges communities’ face, which in turn can help promote health and wellbeing and improve quality of life among children, adolescents, working adults and the elderly across countries, societies and generations. Although social enterprises have been studied as upstream intervention on population health and wellbeing (e.g., women, and elderly), these studies have not been linked to sustainable development frameworks and SDG 3 in particular [29,30,31,32,33]. Therefore, social enterprises present an opportunity to engage businesses as partners in health promotion and achievement of sustainable population health and sustainable and inclusive societies and for all segments of the population (specifically the most disadvantaged).

In addition, SEs can collaborate with public health and contribute to health promotion practice, not only as part of the expected intersectoral collaboration, but with regard to health in all policies – based on “an approach to public policies across sectors that systematically takes into account the health and health systems implications of decisions, seeks synergies, and avoids harmful health impacts, in order to improve population health and health equity” [70].”

According to Bornstein, “what business entrepreneurs are to the economy, social entrepreneurs are to social change.” They are the driven, creative individuals who question the status quo, exploit new opportunities, refuse to give up — and remake the world for the better [71].” And in this spirit, they can be agents of change in public health policy where equitable health for all is a reality.

However, there are several challenges to be considered. Firstly, to date, public health professionals still need to fully embrace businesses (for profit or not) as partners in promoting health and wellbeing, in achievement of SDG 3. Conversely, businesses (especially SEs) do not yet see public health institutions and practitioners as potential partners in their work with communities. Secondly, there is the challenge to get a better understanding of how, and through what activities, SEs will better impact the health and wellbeing of individuals and communities towards attainment of the SDGs and specifically SDG 3. Littlewood and Holt’s point out that, with 17 SDGs and no less than 169 associated targets, understanding how SEs can contribute to the achievement of these goals remains challenging, particularly given the diversity of SE models that exist across the globe [28]. Thirdly, there are those who argue that the SDGs fail to properly acknowledge the potential central role business in general will need to play if they are to be achieved, and more especially the potential contribution of responsible trading, social entrepreneurship and SEs [28,72]. Fourthly, the role of responsible leadership within SEs needs to be better understood. This includes an understanding of how such leadership may impact the drive for successful change as well as activities that might result in sustainable population health. Future public health research needs to include the leadership dimension in parallel with the other aspects of the relation between SEs, health and wellbeing that are already being researched. It has been suggested that responsible leadership (even within SEs) is more likely to “do good” and “not do harm” and to take into consideration the needs of different stakeholders inside and outside the organization [73,74]. This willingness to collaborate will help responsible leaders in SEs to succeed as they help tackle the wicked social problems and face the challenge of creating a more inclusive, healthier, and more sustainable world.

Although inequalities in health are being addressed, still they remain a public health concern in low–, middle–, and high–income countries [75,76]. This is due to the current unequal distribution of social, economic and environmental risks (and hazards) related to where people live, learn, work, play, seek care and spend their time or their individual circumstances. Thus, social enterprise can be an essential partner to local, regional and national governments to make sure that no one is left behind, increasing the chances for the achievement of SDG3. Some argue that SDG3 should not be seen as a single goal for sustainable development, but as means to achieve the three pillars of sustainable development [77]. Social enterprises involvement in improving health and wellbeing represent a shift on the potential role for business in promoting health beyond profit. This is line with the need for partnership for the goals and the need for health in all policies [78,79]. Furthermore, others point out that SDG3 core health targets are either embedded in other goals or influenced by them [80,81,82]. Thus, social enterprises (as part of community businesses) are likely to ensure new ways of stakeholder engagement and partnership for sustainable development goals while contributing to promote health and wellbeing of individuals and communities.
